# Region-wide assessment of the capacity for human nutrition training in West Africa: current situation, challenges, and way forward

**DOI:** 10.3402/gha.v7.23247

**Published:** 2014-01-09

**Authors:** Roger Sodjinou, Nadia Fanou, Lucie Deart, Félicité Tchibindat, Shawn Baker, William Bosu, Fré Pepping, Hélène Delisle, The Regional Nutrition Working Group

**Affiliations:** 1 UNICEF Regional Office for West and Central Africa, Dakar, Senegal; 2 West Africa Health Organization (WAHO), Bobo-Dioulasso, Burkina Faso; 3 Bill and Melinda Gates Foundation, Seattle, WA, USA; 4 Division of Human Nutrition, Wageningen University, The Netherlands; 5 Department of Nutrition, Faculty of Medicine, University of Montreal, Canada

**Keywords:** nutrition, nutrition training, public health nutrition, capacity development, mapping, curriculum development, West Africa

## Abstract

**Background:**

There is a dearth of information on existing nutrition training programs in West Africa. A preliminary step in the process of developing a comprehensive framework to strengthen human capacity for nutrition is to conduct an inventory of existing training programs.

**Objective:**

This study was conducted to provide baseline data on university-level nutrition training programs that exist in the 16 countries in West Africa. It also aimed to identify existing gaps in nutrition training and propose solutions to address them.

**Design:**

Participating institutions were identified based on information provided by in-country key informants, UNICEF offices or through internet searches. Data were collected through semi-structured interviews during on-site visits or through self-administered questionnaires. Simple descriptive and bivariate analyses were performed.

**Results:**

In total, 83 nutrition degree programs comprising 32 B.Sc. programs, 34 M.Sc. programs, and 17 Ph.D. programs were identified in the region. More than half of these programs were in Nigeria. Six countries (Cape Verde, Guinea-Bissau, Liberia, Mali, The Gambia, and Togo) offered no nutrition degree program. The programs in francophone countries were generally established more recently than those in anglophone countries (age: 3.5 years vs. 21.4 years). Programs were predominantly (78%) run by government-supported institutions. They did not provide a comprehensive coverage of all essential aspects of human nutrition. They were heavily oriented to food science (46%), with little emphasis on public health nutrition (24%) or overnutrition (2%). Annual student intakes per program in 2013 ranged from 3 to 262; 7 to 40; and 3 to 10, respectively, for bachelor's, master's, and doctoral programs while the number of graduates produced annually per country ranged from 6 to 271; 3 to 64; and 1 to 18, respectively. External collaboration only existed in 15% of the programs. In-service training programs on nutrition existed in less than half of the countries. The most important needs for improving the quality of existing training programs reported were teaching materials, equipment and infrastructures, funding, libraries and access to advanced technology resources.

**Conclusions:**

There are critical gaps in nutrition training in the West Africa region. The results of the present study underscore the urgent need to invest in nutrition training in West Africa. An expanded set of knowledge, skills, and competencies must be integrated into existing nutrition training curricula. Our study provides a basis for the development of a regional strategy to strengthen human capacity for nutrition across the region.

Adequate nutrition is both a key determinant as well as an outcome of development (1). The prevalence of stunting in children under the age of five is at least 30% in most West African countries. The region has made the least progress in reducing stunting prevalence over the past two decades (2). Further, only two countries in West Africa (Ghana and Liberia) are on track to reduce the prevalence of underweight children by 50% (3). Micronutrient deficiencies are also widespread in the region with devastating consequences on morbidity, mortality, and socioeconomic development (4). At the same time, obesity and diet-related chronic diseases are becoming a serious public health concern in West Africa (5). Yet, the region is facing weak and inadequate institutional capacity as well as a critical shortage of human resource to deliver effective nutrition interventions.

A critical mass of skilled nutrition professionals is needed to design, plan, and monitor nutrition interventions as well as to deliver essential and quality nutrition services at all levels (6). There is an urgent need to strengthen institutional and human capacity for nutrition to achieve nutrition-related Millennium Development Goals (MDGs) and reduce poverty in the West Africa region.

Unlike in other parts of the world, very little effort has been done to improve institutional and human capacity for nutrition in West Africa (7). Most of the nutrition capacity development initiatives have been concentrated on the Eastern and Southern African countries (8). While it is estimated that about 700 nutrition graduates at bachelor's, master's and doctoral level are needed every year in West Africa (7), the current output is around 250 (9). This capacity gap reduces the ability to scale up interventions known to be effective in improving nutrition outcomes. The importance of enhancing human capacity to implement at scale interventions addressing undernutrition has been emphasized in the Scaling Up Nutrition (SUN) framework (10). It should be noted, however, that workforce preparation is only one component of nutrition capacity development, which also includes the system-, organizational- and community-level factors, which are needed to support the performance of nutrition workforce (11).

There is a dearth of published data on functional university-level nutrition training programs in West Africa. In recent years, there has been increasing awareness of the need to generate such data. A preliminary study was conducted a few years ago (12) but it was somewhat limited in scope in terms of the nutrition programs identified. Further, data such as the date of inception, ownership, funding, and focus areas of the programs were not covered. That study (12), as well as the participants of a planning workshop organized in Dakar in 2009, both recommended that a detailed inventory of existing nutrition training programs in the region is urgently needed (9).

The present study was therefore undertaken to provide a comprehensive mapping of the current capacity for nutrition training in West Africa. It also aimed to identify existing gaps in nutrition training and propose solutions to address them. The present inventory was conducted within the framework of the West Africa Nutrition Capacity Development Initiative (WANCDI). This initiative seeks to provide the capacity and skills needed to accelerate progress for nutrition in West Africa. It has been formally endorsed by the Economic Community of West African States (ECOWAS) Assembly of Health Ministers.

## Methods

### Study design

This was a descriptive study of nutrition training programs in 16 West African countries comprising the 15 ECOWAS states plus Mauritania. The West Africa region is divided into three major language groups: anglophone countries (Ghana, Liberia, Nigeria, Sierra Leone, and The Gambia); francophone countries (Benin, Burkina Faso, Côte d'Ivoire, Guinea, Mali, Mauritania, Niger, Senegal, and Togo); and lusophone countries (Cape Verde and Guinea-Bissau).

### Participating institutions

Participating institutions were those offering either a degree-granting program or a non-degree program on human nutrition at the time of data collection. Medical, nursing, or agricultural schools offering nutrition courses as part of their curricula were also included in the study sample. Degree programs heavily weighted to food science, food technology, and biochemistry were excluded.

A preliminary list of institutions was compiled based on information provided by in-country key informants, UNICEF country offices or through internet searches. We continued to update this list until the end of the data collection period.

### Data collection

Data were collected between April and August 2013 using a semi-structured open-ended questionnaire, either self-administered or administered during in-person meetings. A brief introductory email was sent to all of the targeted institutions to explain the objectives of the study and request their participation. The study questionnaire was then sent to the representative of each institution. UNICEF country offices wrote supportive letters to the institutions to encourage response.

During face-to-face meetings with the representatives of institutions, we further explained the objectives of the study to the respondents and answered their questions when needed. Then using the study questionnaire, we gathered information on existing programs, training curricula and their major components: profile of faculty members, students and graduates; funding; facilities; and international collaborations. The study questionnaire also included open-ended questions about respondent's needs for upgrading training curricula and improving the quality of existing training programs. Interviews were conducted by three experienced researchers in the working languages of the respondents. Ten questionnaires administered during a first round of interview served as a pretest. This first round revealed no difficulties in comprehension or response.

Self-administered questionnaires were used when an on-site visit was not possible. In this case, respondents completed the questionnaire and returned it by email. Additional data were collected through literature review when we were unable to make an appointment for on-site visit or obtain information about existing programs. In spite of follow-up reminder emails and calls, we could not obtain responses to all the questions particularly in the self-administered questionnaires.

### Data analysis

Data were entered, double-checked, and cleaned in MS Excel 2010. They were later exported and analyzed in SPSS 14.0. Simple descriptive and bivariate analyses were performed.

The age of training programs was calculated using the following formula: {Age of training program=2013 − Year of inception of the program}. Student intake was defined as the maximum number of participants enrolled for the program. Graduate output referred to the annual number of graduates delivered by the program. A recommended annual output of graduates of 300 bachelor's level, 30 master's level, and 2 doctorate level needed per 5 million inhabitants was used. These figures were adapted from the UNU/IUNS 2007 recommendations (7) and assumed a median professional life span of 10 years (9). Data on populations were derived from the Wikipedia website (13).

### Ethical considerations

No ethical approval was sought as the study did not involve data collection on human subjects. All respondents were fully informed about the objectives of the assessment. They gave their full verbal consent prior to participating in the study.

## Results

**Fig. 1:**
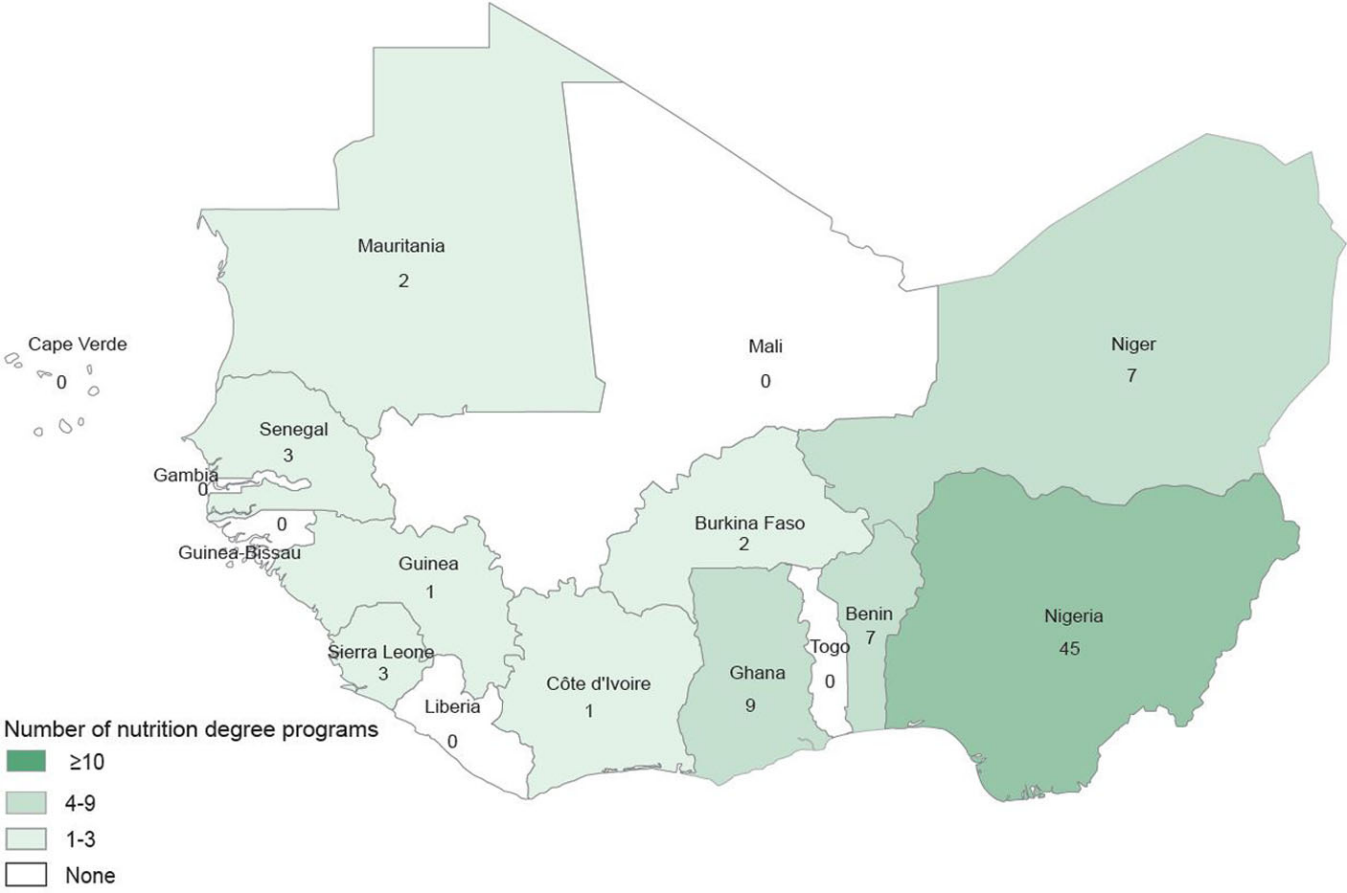
Summary of existing nutrition degree programs in West Africa.

Data on 234 (76%) of the 306 training programs were collected using direct interviews or self-administered questionnaires (Table 1). The training programs were organized by a total of 114 training institutions. Of the total of 83 nutrition degree programs offered in 10 countries in the region, 47 were in Nigeria alone (Fig. 1 and Table 2). The detailed list of nutrition training programs is available upon request.

**Table 1:** Participating institutions and data collection methods

Language usage	Countries	Number of participating institutions	Number of training programs assessed	Methods used to collect information about training programs		

				Direct interview	Self-administered questionnaire	Literature review and internet search
French	Benin	2	13	4	9	0
	Burkina Faso	7	26	26	0	0
	Côte d'Ivoire	4	9	7	2	0
	Guinea	12	24	19	5	0
	Mali	13	18	0	0	18
	Mauritania	5	15	6	9	0
	Niger	23	57	21	23	13
	Senegal	9	34	16	18	0
	Togo	2	2	1	1	0
	Sub-total	77	198	103	67	28
English	Ghana	5	15	13	1	1
	Liberia	2	2	2	0	0
	Nigeria	24	68	16	10	42
	Sierra Leone	5	22	17	5	0
	The Gambia	1	1	0	0	1
	Sub-total	37	108	48	16	44
Total		114	306	151	83	72

**Table 2:** Nutrition degree programs operating in the West Africa region

	Bachelor's degree					Master's degree					Doctorate degree				
			
	Number of programs	Mean age	Mean duration	Ownership		Number of programs	Mean age	Mean duration	Ownership		Number of programs	Mean age	Mean duration	Ownership	
			
Country				Public	Private				Public	Private				Public	Private
Benin	2	4.5	36	2	0	4	6.3	21.5	4	0	1	6	48	1	0
Burkina Faso	1	3	36	1	0	1	5	24	1	0	0	–	–	–	–
Côte d'Ivoire	0	–	–	–	–	1	3	24	1	0	0	–	–	–	–
Ghana	4	30.5	48	4	0	4	7	24	4	0	1	1	36	1	0
Guinea	1	1	48	1	0	0	–	–	–	–	0	–	–	–	–
Mauritania	1	4	36	1	0	1	4	24	1	0	0	–	–	–	–
Niger	6	7	36	2	4	2	1	24	1	1	0	–	–	–	–
Nigeria	15	20.7	47.2	10	5	18	22.7	22.6	15	3	14	22.8	36	9	5
Senegal	0	–	–	–	–	2	2	24	2	0	1	15	48	1	0
Sierra Leone	2	23	48	2	0	1	23	24	1	0	0	–	–	–	–
Total	32	–	–	23	9	34	–	–	30	4	17	–	–	12	5

–: non-existing

### Undergraduate nutrition degree programs

In total, 32 undergraduate nutrition programs were offered in eight of the 16 countries surveyed (Table 2). Three countries accounted for 25 of these 32 undergraduate programs – Nigeria (15 programs), Niger (six programs), and Ghana (four programs). Eight (mostly lusophone and francophone) countries – Cape Verde, Côte d'Ivoire, Guinea-Bissau, Liberia, Mali, Senegal, The Gambia, and Togo – did not have any undergraduate nutrition programs.

There was a marked difference between francophone and anglophone countries with respect to the age of training programs (Table 2). Francophone country training programs were much more recent than those of anglophone countries (mean age: 4.9 vs. 24.1 years). The oldest undergraduate nutrition programs in the region were established in Legon (Ghana) in 1960 and Ibadan (Nigeria) in 1963.

The basic requirement for admission was a college degree in biology, biochemistry or a related field. The mean duration of undergraduate nutrition programs was shorter in francophone than in anglophone countries (38.0 vs. 47.4 months). Nearly all (10/11) programs in francophone countries were of 3 years duration on a full-time basis. In contrast, nearly all (20/21) programs in anglophone countries were 4 years duration (Table 2).

On average, student intakes were much higher in anglophone countries than in francophone countries (93.9 vs. 29.5 respectively; Table 3). The highest annual intakes were observed in Nigeria (940 students) and Ghana (275 students). Enrolments were relatively modest in francophone countries, with a range of 25–125 for Benin, Burkina Faso, Guinea, Mauritania, and Niger (Table 3).

**Table 3:** Number of nutrition graduates and training intake by country and degree program

		Graduates (2012)						Annual intakes of students (2013)					
			
		Bachelor's degree		Master's degree		Doctorate		Bachelor's degree		Master's degree		Doctorate	
							
West Africa countries	Population (millions of inhabitants)^a^	Needs	Number of graduates	Needs	Number of graduates	Needs	Number of graduates	Range (per program)	Total (per country)	Range (per program)	Total (per country)	Range (per program)	Total (per country)
Benin	9.7	58	6	6	42	3	1	20–25	45	10–30	75	3	3
Burkina Faso	17.3	104	ND	10	12	5	0	25	25	25	25	–	–
Cape Verde	0.5	3	0	0	0	0	0	–	–	–	–	–	–
Côte d'Ivoire	23.9	144	0	14	12	7	0	–	–	ND	ND	–	–
Gambia	1.8	11	0	1	0	1	0	–	–	–	–	–	–
Ghana	26.4	159	107	16	16	8	1	20–100	275	10–15		ND	ND
Guinea	11.9	71	ND	7	0	4	0	50	50	–	–	–	–
Guinea-Bissau	1.7	10	0	1	0	1	0	–	–	–	–	–	–
Liberia	3.9	23	0	2	0	1	0	–	–	–	–	–	–
Mali	16.7	100	0	10	10	5	0	–	–	–	–	–	–
Mauritania	3.5	21	17	2	0	1	0	30	30	30	20	–	–
Niger	17.5	105	86	10	0	5	0	15–45	125	20–25	45	–	–
Nigeria	177.1	1063	271	106	64	53	18	3–262	940	10–40	177	5–10	25
Senegal	13.6	81	0	8	3	4	1	–	–	7–20	27	9	9
Sierra Leone	5.8	35	30	3	15	2	0	60	120	30	30	–	–
Togo	6.7	40	0	4	0	2	0	–	–	–	–	–	–
Total	338.0	2028	517	203	174	101	21	3–262	1,610	7–40	471	3–10	37

– : non-existing; ND: no data.

a Data were derived from the Wikipedia website (8).

Based on available data, we estimated that 517 Bachelor's-level graduates were produced from six countries in the region in 2012, which is far below the recommended level of 2,028 graduates per year (Table 3). The highest outputs were observed in Nigeria (271), Ghana (107), and Niger (86).

Based on data from 17 out of the 32 undergraduate programs, we estimated that the average number of teaching staff per program was 14.4 (Table 4). A large share of 67% (163/245) of the teaching members was full-time staff. Nearly three quarters (73%, 178/245) of them had a doctorate qualification.

**Table 4:** Characteristics of teaching staff

		Bachelor's degree (*n*=17)		Master's degree (*n*=25)		Doctorate (*n*=10)	
				
Highest degree		Full-time^a^	External^b^	Full-time	External	Full-time	External
Qualification of teaching members	Doctorate or above	122	56	218	61	101	22
	Master's degree or equivalent	32	13	59	76	0	0
	Bachelor's degree or equivalent	9	13	0	0	0	0
Total teaching staff per program		163	82	277	137	101	22
Average number of teaching staff per program		9.6	4.8	11.1	5.5	10.1	2.2

a Full-time: teaching member employed on a full-time basis by the department offering the training program.

b External: Lecturer working in another department or university but teaching some courses.

**Fig. 2:**
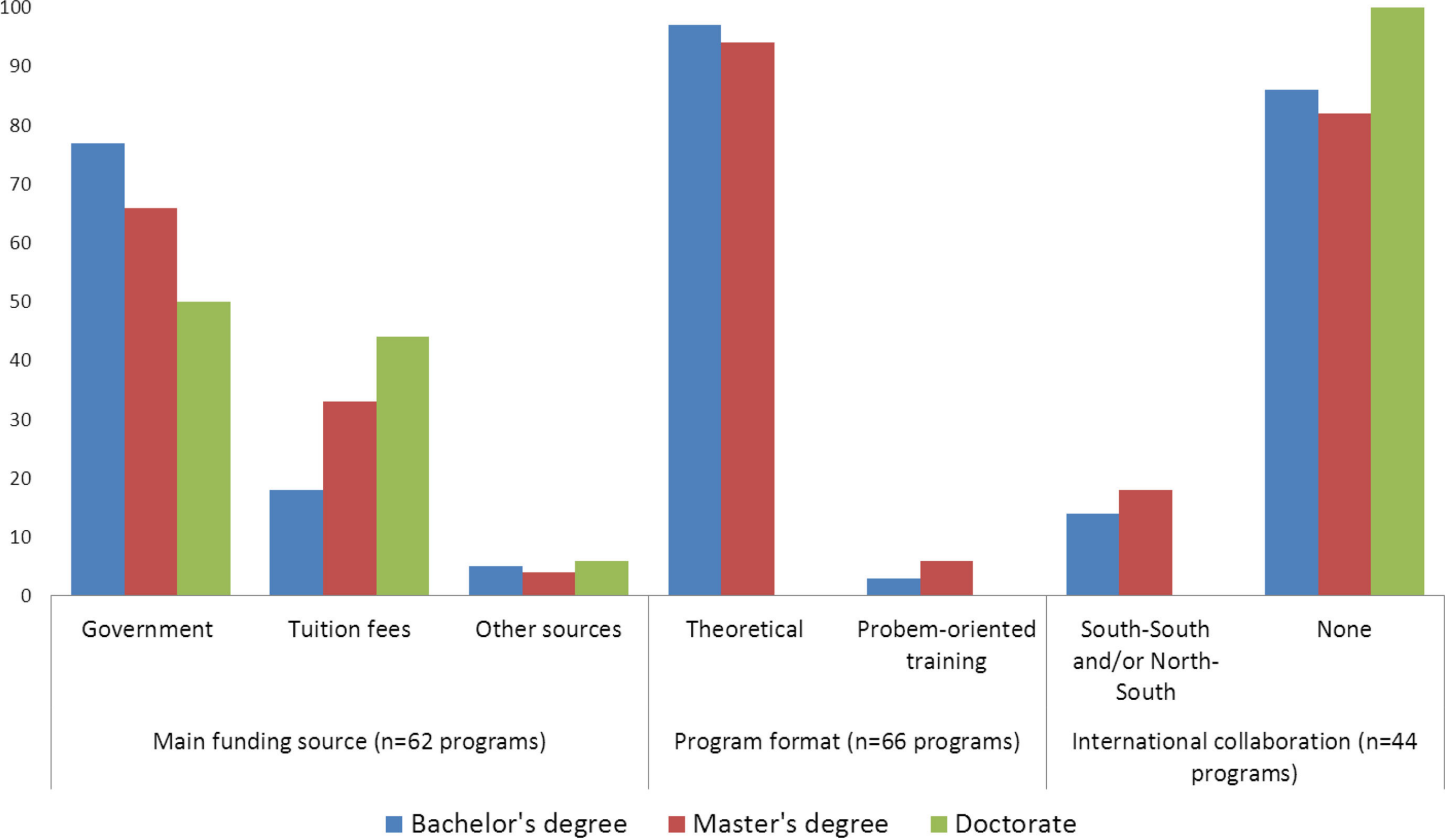
Characteristics of nutrition training programs.

**Fig. 3:**
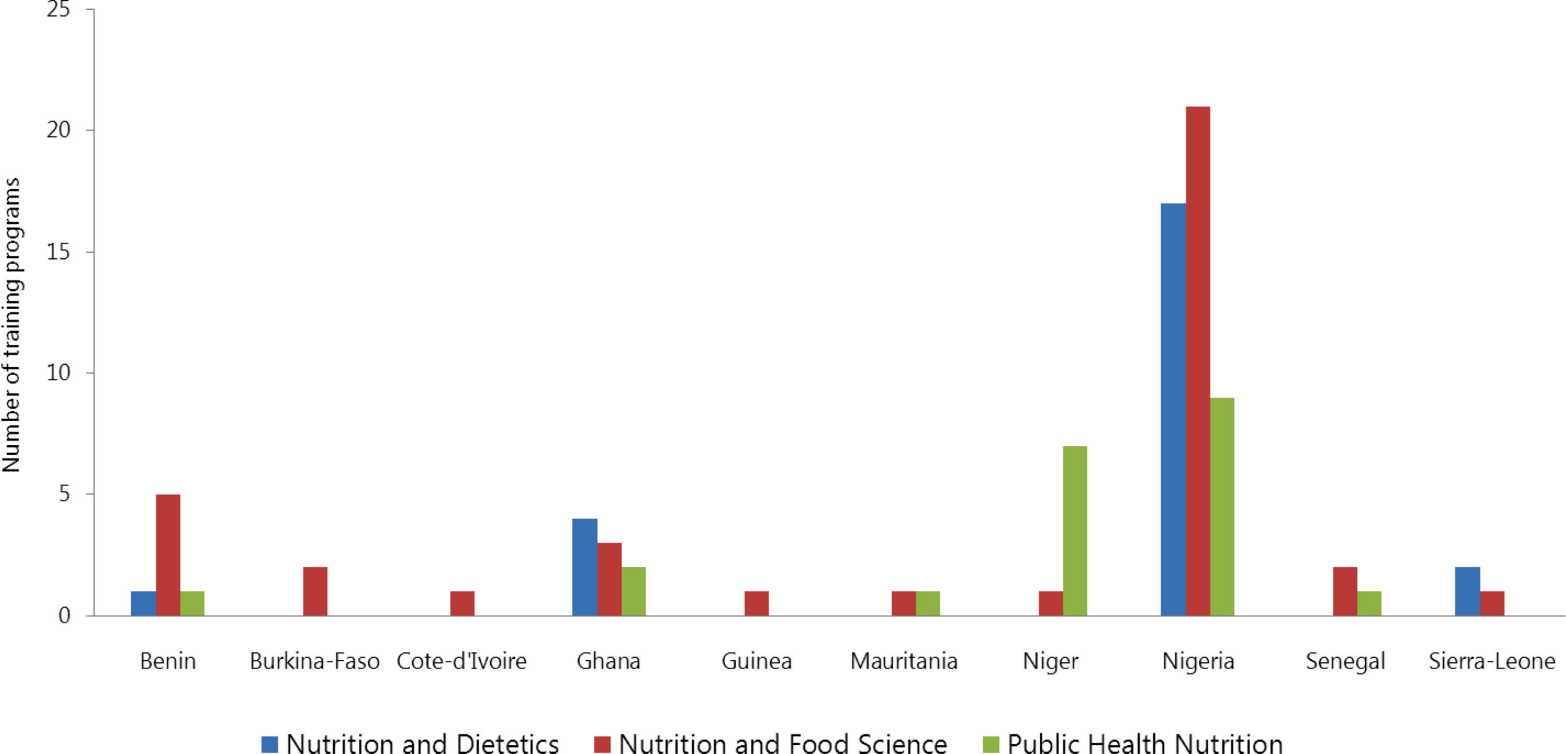
Focus areas of existing nutrition degree programs.

In nearly all of the training programs (97%, 31/32 programs), the teaching format was predominantly theory-based, with less time devoted to practical hands-on or problem-oriented training (Fig. 2). In general, training curricula included courses on basic sciences (organic chemistry, biochemistry, physiology, microbiology, cell biology, statistics, enzymology, etc.), food science (food processing, food chemistry, food microbiology, food composition, food quality control, food legislation, etc.), and nutrition (nutrients and their metabolism, nutritional requirements, nutritional physiology and biochemistry, etc.). Existing nutrition training programs did not provide comprehensive coverage of all essential aspects of human nutrition (Fig. 3). About one-third of undergraduate training programs was heavily weighted to basic nutrition and food science (34%, 11/32), while one-fifth was oriented to public health nutrition (22%, 7/32). Courses on public health aspects of maternal and child nutrition, nutrition policies and programs, physical activity, overnutrition, and non-communicable diseases were covered marginally.

Most of the programs (72%, 23/32 programs) were run by state-owned institutions. Nine private institutions (four in Niger and five in Nigeria) offered undergraduate nutrition training in the region (Table 2). Programs managed by private institutions were much more recent than those of public institutions (mean age: 6.8 vs. 21.4 years). State-owned programs relied mostly (77%) on government subsidies as their main funding source while private institutions relied mostly on tuition fees (Fig. 2).

There were few institutions engaged in partnership with external organizations for nutrition training. Only 14% (2/14) of the program's bachelor level were involved in international collaboration (Fig. 1).

### Master's programs in nutrition

In total, 34 programs were offered in 9 of 16 the countries (Table 2). The number of master's programs in each of these countries ranged from 1 to 18. Countries lacking master's level nutrition training programs were Cape Verde, Guinea, Guinea-Bissau, Liberia, Mali, The Gambia, and Togo.

As with the undergraduate programs, francophone master's programs were established more recently than those of anglophone countries (mean age: 4 vs. 20 years, respectively).

A general requirement for admission for most of the programs was a bachelor's or license degree in nutrition or an acceptable equivalent from an approved university. In addition, some programs required some years of professional experience in nutrition. In anglophone countries, a second-class upper division (equivalent of 60–70%) was generally required for admission to MPhil degree.

The majority of the programs (85%, 29/34) required 2 years of full-time study. Four universities in Nigeria offered an 18-month program while a program in Benin lasted 14 months.

The total capacity for master's program was over 471. The annual intakes per program ranged from 7 to 40 (Table 3). At country level, the highest intakes were observed in Nigeria (177 participants a year). Based on available data, 174 graduates at master's level were produced each year in the region. This is far below the recommended level of 203 (Table 3). The annual number of master's graduates produced per country ranged from three in Senegal to 64 in Nigeria in 2012 (Table 3).

Based on data from 25 master's level programs, we estimated that the average number of teaching staff for master's program was 17 per program (Table 4). Of the 414 teaching members involved in training at this level, two-thirds were full-time staff, 279 (67%) had a doctorate qualification, while 135 (33%) had a master's qualification.

The teaching format for master's degree programs was mainly (94%, 32/34) based on the traditional teaching format (Fig. 2). All of the master's programs were by coursework. There were generally two distinct graduation pathways: master's thesis and professional master's programs. Students choosing the first option carry out a research project. The second option allows students to perform a project in a professional setting and write a report. MPhil programs were mainly by research, followed by a dissertation. Training curricula were oriented to food science in most of the cases (18/34) while some (20%, 7/34) of the programs were related to nutrition and dietetics. Public health nutrition was the focus for 27% (9/34) of the programs.

Most of the programs (88%, 30/34) were run by government-owned institutions (Table 2). The remaining four were private institutions (one in Niger and three in Nigeria). Government subsidies were the main funding source for most (66%) of the programs (Fig. 2).

International collaboration for master training in nutrition was observed in some 25% of the programs (6/23). Existing partnerships were mainly for nutrition research.

### Doctorate degree programs in nutrition

In total, 17 doctoral degree programs existed in four countries – Nigeria, Benin, Ghana, and Senegal with 14 programs in Nigeria alone (Table 2).

A general requirement for admission for doctorate programs was an MPhil or master's degree in nutrition or in a related field. Most programs (88%, 15/17) required 3 years for students to graduate. However, the duration was 48 months in Benin and Ghana. In practice, it takes longer than that for students to graduate. Doctorate programs were mainly by research. Most (53%, 9/17) of the doctorate programs assessed were on nutrition and food science. Little emphasis was put on public health nutrition (29% only, 5/17) or nutrition and dietetics (18% only, 3/17).

The total annual student intake at doctorate level was over 37 in the region. The highest intakes were observed in Nigeria (on average 25 Ph.D. candidates a year). The intakes per program ranged from three in Benin to 10 in Nigeria (Table 3).

Based on available data, 21 graduates at doctorate level were produced in the region, which is well below the recommended level of 101 (Table 3). The majority (86%, 18/21) of these graduates was produced in Nigeria.

From 10 doctoral level programs, we calculated the average number of teaching staff per program as 12.3 ( 4). Of the 123 teaching members involved in doctorate training, 82% (101/123) were full-time staff and all of them had a doctorate qualification.

Most (88%, 15/17) of the programs were run by public, government-owned institutions. However, there were five doctorate programs in Nigeria organized by private institutions (Table 2).

### Short-term nutrition courses

There were several non-degree-granting short courses offered in Benin, Burkina Faso, Niger, Nigeria, Senegal, Sierra Leone, and The Gambia. They were aimed at in-service professionals who would like to refresh or upgrade their knowledge in nutrition. These programs were mainly on-site and their duration ranged from 6 days to 9 weeks. Certificates of attendance are awarded to candidates upon successful completion of the training program. Topics covered include treatment of acute malnutrition, prevention of the double burden of malnutrition, nutrition education, and promotion of infant and young child feeding practices.

### Diploma courses in nutrition

Several universities in Nigeria offered postgraduate diploma courses in nutrition as part of their efforts to provide in-service training to professionals. Their duration varied between 12 and 18 months. Candidates applying for postgraduate diploma programs should possess the minimum entry requirements for admission into the first-degree programs in nutrition, agriculture or a related field. Candidates admitted to postgraduate diploma courses were generally required to undergo a period of professional attachment or internship in the field of nutrition during the course. Postgraduate diploma holders may be qualified for master's degree admission.

### Nutrition courses in nursing and medical schools

We found that health and agricultural professionals received very little nutrition courses as part of their curricula. In most of the cases, these courses were limited both in terms of coverage and duration. The nutrition courses offered in medical and nursing schools were mainly on clinical nutrition, nutrition-related disorders, and dietetics.

### Reported needs for upgrading the quality of existing programs

Participating institutions mentioned some constraints which limit the effective implementation of their training curricula. They also expressed their needs for improving the quality of their training programs. The most important reported needs were teaching materials, equipment and infrastructures, funding, libraries, and access to advanced technology resources (Table 5).

**Table 5:** Perceived needs (*n*=44 institutions)

Perceived needs	%
Upgrade nutrition training curricula to the latest developments	45.5
Upgrade and empower human resource for nutrition training	27.3
Improve equipment and infrastructure	27.3
Improve funding	22.7
Development of institutional collaboration (internal/external) for nutrition training	18.2
Access to library and advanced technology resources (computers, internet, etc.)	13.6
Review and harmonize training curricula to meet national or regional needs	13.6
More exposure of students and more courses emphasizing on practical knowledge	11.4

## Discussion

In this descriptive study, we assessed the current capacity for nutrition training in the West Africa region in some detail. We also assessed the challenges that existing nutrition training programs are facing and explored ways to address them.

Our study revealed that 83 nutrition degree programs are offered in 10 countries in West Africa, of which more than half were in Nigeria alone. The dominance of Nigeria is not unexpected, given its population. More than one-third of the identified programs started less than 10 years ago, suggesting an increasing priority to nutrition training. We noticed an increasing role of private universities in Niger and Nigeria. It is gratifying that some of the progress has been observed in countries such as Niger, Mauritania, and Burkina Faso with the greatest nutrition burdens.

Until recently, undergraduate nutrition training was not common in francophone countries of West Africa, partly because nutrition had not been an autonomous university discipline in the French academic system. The recent introduction of the triple structure model of bachelor's, master's, and doctoral degree has enabled the inception of undergraduate nutrition training programs in francophone West Africa. The rapid expansion of undergraduate nutrition programs in Niger deserves mention. The cyclical nutrition crises have raised awareness on the urgent need to train more highly skilled nutrition professionals to deliver emergency nutrition interventions. Unfortunately, there has been a slower response in strengthening nutrition human resource capacity in Mali.

Despite the progress in nutrition training observed, several challenges were identified. We did not find any nutrition degree program in six countries – Cape Verde, Guinea-Bissau, Liberia, Mali, The Gambia, and Togo. Other challenges included training curricula which were not aligned to regional nutrition priorities, teaching methods that were very didactic and did not prepare students to tackle real-life problems, insufficient number of trainers, limited production of nutrition graduates and low levels of institutional collaboration. It is not clear if this reflects a more general weakness in high-level training in Africa. For many years, higher education in Africa has received far less attention than primary and secondary schooling, perceived by African governments as a driver of economic growth and poverty reduction (14). There is an urgent need to strengthen institutional capacity for tertiary-level nutrition training in West Africa.

A high proportion of existing nutrition degree programs did not cover all essential aspects of human nutrition, with some being heavily oriented to food science. The custom of having both nutrition and food science combined in one degree program should be avoided. There is the need to re-orient courses to adequately cover current and emerging issues such as public health nutrition, emergency nutrition, and community-based management of acute malnutrition. There is also a need to emphasize overnutrition and chronic non-communicable diseases as they are on rise in the region (15–17). An expanded set of knowledge, skills, and competencies must be integrated into existing nutrition training curricula to adequately prepare nutrition graduates to address the prevailing nutrition problems in the region. Several nutrition scientists have stressed the need to have competency-based training in nutrition in West Africa (18, 19). The competency framework for capacity development in public health nutrition proposed by Hughes et al. (20) could be adapted to the West African context for master's programs.

As nutrition gains momentum in the region, nutrition graduates will have to drive nutrition processes, play a leading role, manage nutrition portfolio, and influence development agenda in favor of nutrition. Therefore, they need cross-cutting skills that go beyond technical competencies. It is therefore essential to incorporate courses on leadership, management, communication, and advocacy into existing postgraduate curricula. Examples of successful initiatives on nutrition leadership exist on the continent (e.g. African Nutrition Leadership Program in South Africa and the *Programme de Leadership Africain en Nutrition* in Morocco).

The ongoing debate on the post-2015 development agenda offers the opportunity to reflect on what could be the nutrition training curriculum standards in the West Africa region. The UNICEF conceptual framework of undernutrition underscores that achieving nutrition security is a task that involves sectors such as agriculture, health, education, water, sanitation, hygiene, social protection, and nutrition (21). There is a need for standard curricula for pre-service and in-service nutrition training that cover all these sectors. Technical nutritionists, health workers, agricultural specialists, and other cadre should be equipped with knowledge to tackle multidimensional nutrition problems in the region.

We found that institutional collaboration for nutrition training (whether it is South–South or North–South) is not well developed. The need for closer ties and collaboration between francophone and anglophone countries in Africa has been stressed previously (19). There are many benefits and incentives for universities in West Africa to engage in external collaboration for nutrition training. These include reducing human resource shortages, facilitating the exchange and mobility of expertise across the region, gaining an opportunity to update and upgrade training curricula, and taking advantage of reduced cost of use of new information technologies.

Overall, our results underscore the urgent need to harmonize nutrition curricula at each level of training across the West Africa region and to define a set of minimum standards to qualify as a nutritionist (8, 18). WANCDI will work with the West Africa Health Organization (WAHO), which has experience in harmonizing the curricula for medical training in the region (22), to ensure a formal endorsement of the revised nutrition training curricula.

There are several limitations of this study. First, some respondents did not provide answers to all of the questions. Second, it was difficult to cover the questions on the actual graduates, as most of the surveyed institutions did not have a system in place to track them. Third, although efforts were made to identify all of the nutrition degree programs that were operating in the region, some of them might have been omitted in this inventory, particularly the medical/ nursing schools offering nutrition courses. Finally, the quality of the programs was not assessed with the survey tools, although the areas emphasized by various programs were identified.

These limitations notwithstanding, our study has several strengths. This is the first region-wide assessment of the current capacity for nutrition training in West Africa. Our study provides useful baseline data on the capacity for nutrition training that currently exists in the region. Further, the majority of the data of the present study was obtained through direct interviews with stakeholders. Our study provides a practical basis for the development of a regional strategy to strengthen human capacity for nutrition across the region.

## Conclusions

Our study has shown that degree nutrition programs are generally not well developed in the West African region, particularly in francophone and lusophone countries. Training curricula and teaching methods are not well aligned to the prevailing and emerging nutrition problems in the region. There are considerable human and financial resource challenges which hinder faculty development, research, and training outputs. There is a need for greater collaboration between nutrition training institutions. It is also essential for governments, international organizations, and bilateral donors to increase their investments and support for nutrition and further support nutrition capacity development in the region. Our study provides a practical basis for expanding and improving the quality of nutrition training programs in West Africa.
